# Senescent melanocytes driven by glycolytic changes are characterized by melanosome transport dysfunction

**DOI:** 10.7150/thno.84912

**Published:** 2023-07-03

**Authors:** Young Joon Park, Jin Cheol Kim, Yeongeun Kim, Yul Hee Kim, Soon Sang Park, Charlotte Muther, Agnès Tessier, Gimyung Lee, Gaëlle Gendronneau, Sandra Forestier, Youcef Ben-Khalifa, Tae Jun Park, Hee Young Kang

**Affiliations:** 1 Department of Dermatology, Ajou University School of Medicine, Suwon, Korea.; 2 Innovation Research and Development, Chanel Parfums Beauté, Pantin, France.; 3 Department of Biochemistry and Molecular Biology, Ajou University School of Medicine, Suwon, Korea.; 4 Inflamm-Aging Translational Research Center, Ajou University School of Medicine, Suwon, Korea.

**Keywords:** glycolysis, melanocyte, melanosome, senescence, single-cell RNA sequencing, photoaging

## Abstract

**Rationale:** Senescent melanocytes accumulate in photoaged skin and are closely related to skin aging. A better understanding of the molecular characteristics of senescent melanocytes may be the key to controlling skin aging.

**Methods:** We have developed an *in vitro* model of senescence in melanocytes using UV irradiation and investigated the functional characteristics and molecular mechanisms underlying senescence in UV-irradiated melanocytes.

**Results:** We have highlighted that *in vitro* senescent melanocytes are characterized by melanosome transport dysfunction resulting in melanin accumulation. The defective melanosome transport was confirmed with the ultrastructural characterization of both *in vitro* UV-induced senescent melanocytes and *in vivo* melanocytes of hypopigmented aging skin. A single-cell transcriptomic analysis revealed that the glycolytic metabolism pathway appeared to be significantly upregulated in most senescent phenotypes. Furthermore, the inhibition of glycolysis by pharmacological compounds mitigates the pro-aging effects of melanocytes senescence, suggesting that alterations in cellular glucose metabolism act as a driving force for senescence in melanocytes.

**Conclusion:** These results demonstrate that senescent melanocytes are characterized by glycolytic metabolism changes and a defective melanosome transport process, which may be related to impaired mitochondrial function, highlighting the importance of metabolic reprogramming in regulating melanocyte senescence.

## Introduction

Senescent cells accumulate in several tissues during the aging process, including the skin, and contribute to the functional decline of the skin *via* the senescence-associated secretory phenotype (SASP), which is characterized by the secretion of pro-inflammatory factors 1. Most studies have focused on the dermal compartment, fibroblasts being considered as the major cell type responsible for senescence in skin 2-8. The senescent fibroblasts have been implicated in the development of the skin-aging pigmentation, including solar lentigo and melasma 2, 3, 7, 8. Notably, these fibroblasts exhibit elevated expression levels of pro-melanogenic factors 4-6. However, little is known about the role of melanocytes in senescence during aging. Recently, several studies have highlighted the expression of senescent markers by melanocytes and their accumulation in aged skin 9-11. Indeed, melanocytes appear as the main senescent cells in the epidermis. Senescent melanocytes were observed in the photo-exposed skin of middle-aged individuals and were found to increase sequentially with age, differing from dermal senescent fibroblasts observed regardless of age 10. A recent study has suggested that senescent melanocytes may act as an active driver of skin aging. Senescent melanocytes can affect neighboring cells *via* paracrine telomere dysfunctions of keratinocytes and fibroblasts, contributing to skin aging 12. These cells are also thought to be involved in the pathogenic process that leads to hypopigmentation. Indeed, p16^INK4A^-expressing melanocytes have been found in hypopigmented aging skin, i.e., idiopathic guttate hypomelanosis (IGH) and perilesional vitiligo skin 9, 13, 14.

The differences in molecular mechanism alterations between various types of cells undergoing senescence offer a possible cell-type-specific target for aging modulation. Melanocytes are likely to undergo exceptional alterations while undergoing senescence given their specific role in ultraviolet (UV) protection 11, 15. The melanogenesis process have diverse functions, including cytotoxic, genotoxic, mutagenic, and immunosuppressive activities 16-19. From a metabolic perspective, melanogenesis can affect glycolysis 17 including stimulation of aerobic glycolysis by L-DOPA 18. Thus, a better understanding of the molecular characteristics of senescent melanocytes under chronic UV exposure will provide a new mechanistic basis for understanding the process of senescence.

Here, we investigated the functional characteristics and molecular mechanisms underlying senescence in UV-irradiated melanocytes. UV-induced senescent melanocytes (UVSM) and the melanocytes of hypopigmented aging skin showed significant dysfunction of the melanosome transport process. A single-cell transcriptomic analysis revealed that increased glycolysis is interlinked with melanocyte senescence. Furthermore, the pharmacological inhibition of glycolysis mitigated the pro-aging effects of melanocyte senescence, suggesting that cellular glucose metabolism alterations may act as a force that drives senescence in melanocytes.

## Results

### Chronic UV-exposure induces melanocyte senescence

**Figure 1:**
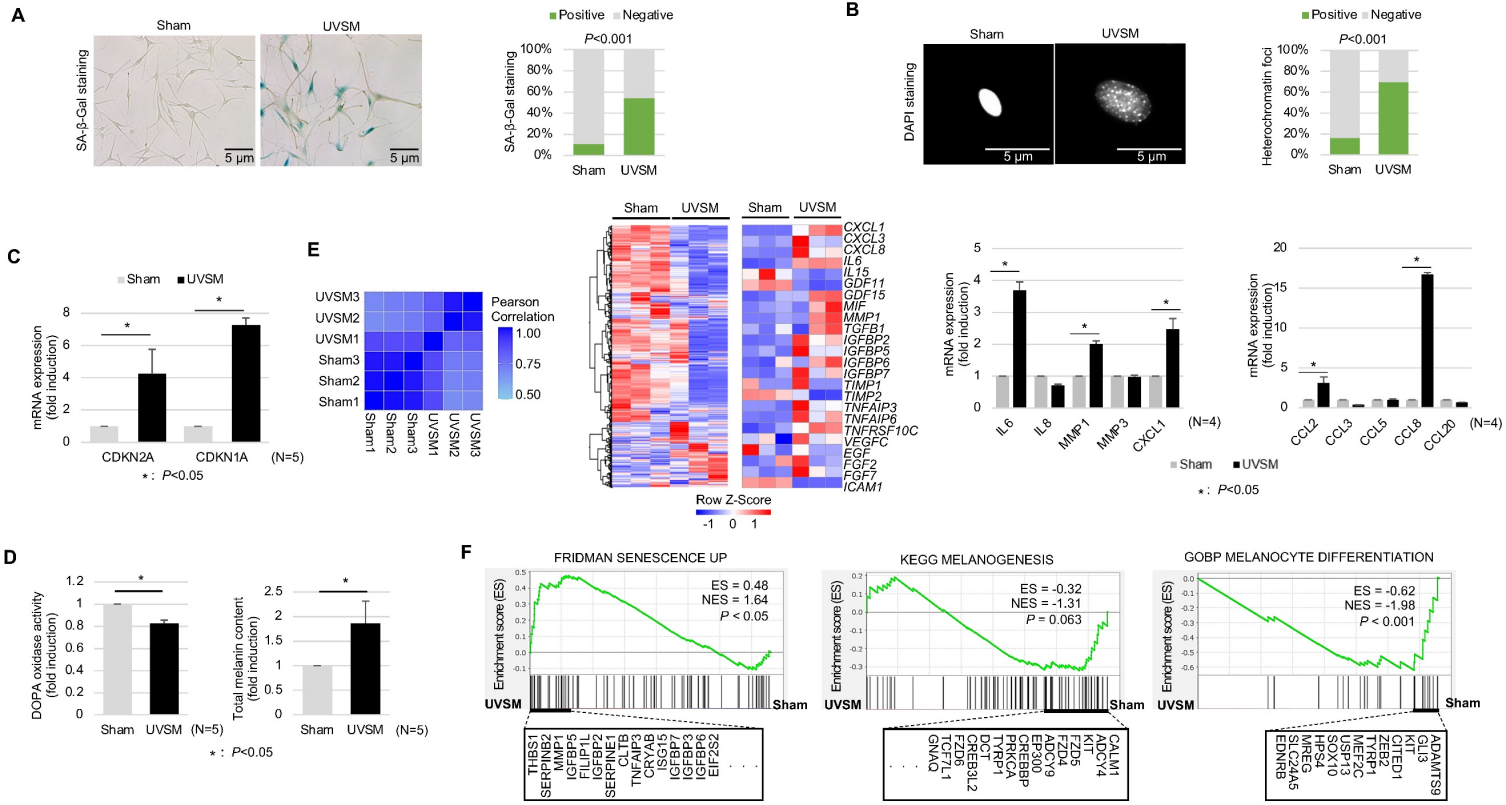
** Characterization of UVB-induced senescent melanocytes.** Normal primary melanocytes were irradiated with 60 mJ/cm^2^ UVB for 4 consecutive days and then cultured for ten days following UV irradiation and cellular senescence was analyzed according to SA-β-Gal staining (A), heterochromatin foci formation (B) and p16^INK4A^/p21^Waf1^ mRNA expression levels (C). (D) Melanin contents and DOPA oxidase activity levels in UVB-induced senescent melanocytes. Similarity metrics and differential gene expression profiling including SASP of sham-irradiated and UVB-induced senescent melanocytes (UVSM) detected by RNA seq analysis (E, left and middle panels). Expression levels of the mRNA of SASPs as analyzed by real-time PCR (E, right panel). (F) GSEA was performed using the “FRIDMAN senescence,” “KEGG melanogenesis” and “GOBP melanocyte differentiation” gene sets to demonstrate the sequential transition in gene expression from sham-irradiated to UVB-induced senescent melanocytes. Statistical significances were assessed using chi-square test for categorical data (A and B) and Mann-Whitney U test for numerical data (C, D, E, and F) in independent two group comparison.

To understand the molecular characteristics of senescent melanocytes, we designed an UV-induced senescence model using isolated normal primary human melanocytes. Melanocytes were treated with UVB at 60 mJ/cm^2^ once daily consecutively for four days and were maintained for ten days before the evaluation. The UV-irradiated melanocytes showed a significant increase in senescence-associated (SA)-β-galactosidase staining, a representative senescence marker 20, and senescence-associated heterochromatic foci formation **(Figure 1A-B)**. Cellular senescence was investigated further by assessing the cell growth rate and, more specifically, the expression levels of the cell cycle regulators *CDKN2A* and* CDKN1A* encoding for cyclin-dependent kinase inhibitors. Significant inductions of *CDKN2A and CDKN1A* were noted in the UV-irradiated melanocytes **(Figure 1C)**. Several reports have shown that increased melanin contents and SASP expression are characteristics of senescent melanocytes 21. Indeed, the melanin content was increased in the UV-irradiated melanocytes here **(Figure 1D, right panel)**. Furthermore, the transcriptomic profiles of the UV-irradiated melanocytes showed completely different gene expression profiling from sham-irradiated melanocytes with an increased SASP gene expression (**Figure 1E, middle panel**). Furthermore, mRNA expression levels of gene-encoding SASP factors, especially IL-6, MMP1, CCL2, CXCL1, CCL2, and CCL8, were significantly increased in UV-irradiated melanocytes **(Figure 1E, right panel)**. Considering all these results, persistent UV exposure can induce senescence of melanocytes, termed UVSM in this study. Interestingly, the melanin content was significantly increased in UVSM, as indicated in **Figure 1D (right panel)**; however, the transcriptomic profiles of UVSM showed lower gene set expression levels during melanogenesis and melanocyte differentiation compared to sham-irradiated melanocytes **(Figure 1F).** Moreover, DOPA oxidase activity was not increased in UVSM compared to sham-irradiated melanocytes **(Figure 1D, left panel)**. These data led us to hypothesize that the increased melanin content observed in UVSM was not caused by an increase in melanin synthesis but rather by a different mechanism.

### Senescent melanocytes exhibit increased melanin contents without any increase of melanogenesis as well as excessive melanosome accumulation caused by a defective melanosome transport process

**Figure 2:**
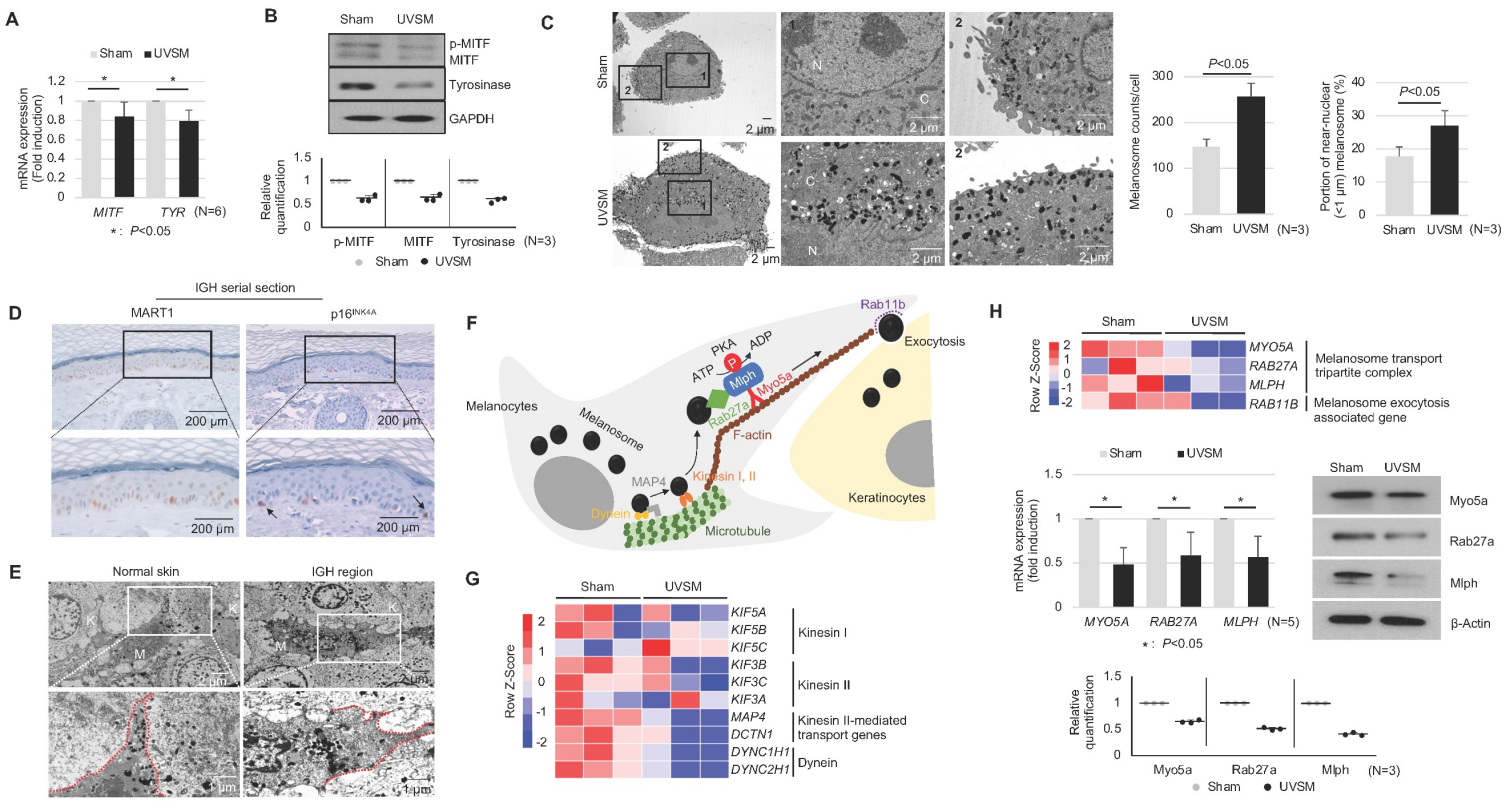
** Senescent melanocytes exhibit melanosome accumulation with defective melanosome transport.** Normal primary melanocytes were irradiated with UVB and cultured for ten days, and MITF/Tyrosinase mRNA (A) and protein (B) were analyzed by real-time PCR and western blotting, respectively. Quantitative results of western blotting were described with dot graph (B, lower panel). (C) Sham-irradiated and UVB-induced senescent melanocytes were analyzed by EM (left panel). '1' and '2' indicate fields observed with a higher magnification. 'N' and 'C' indicate nucleus and cytoplasm, respectively. The total melanosome count and melanosome distribution were analyzed and are presented as a bar graph (right panel). (D) IHC analyses of p16^INK4A^ and MART1 were performed in series sections of IGH patients. Arrows indicates p16^INK4A^-positivemelanocytes. (E) EM analysis of IGH patient skin samples. 'M' and 'K' indicate melanocytes and keratinocytes, respectively. Red lines indicate the melanocyte boundary. (F) A schematic image of the melanosome transport process in melanocytes. (G) Heatmap showing log2 (-fold) changes in the expression levels of kinesin, and dynein as obtained from mRNA-seq (sham *vs.* UVB-induced senescent melanocytes). (H) The melanosome transport tripartite complex gene expression level was analyzed by mRNA seq (upper panel), real-time PCR and western blot (middle panel) analyses. Statistical significances were assessed using Mann-Whitney U test for numerical data (A, C, and H) in independent two group comparison.

To elucidate the functional characteristics of UVSM, the expressions of key genes and proteins in melanogenesis were evaluated. Although the melanin contents were increased significantly in UVSM cases, the mRNA expression levels of *MITF* and *TYR* were significantly downregulated in UVSM **(Figure 2A)**. This was confirmed at the protein level, with reduced levels of MITF and tyrosinase in the UVSM **(Figure 2B)**. To investigate the discrepancy between the decreased melanogenesis and increased melanin content, we undertook an electron microscopy (EM) evaluation of sham-irradiated melanocytes and UVSM. The total melanosome count was increased in the UVSM, as expected **(Figure 2C, middle panel)**. The distribution of melanosomes, however, was significantly different between the sham-irradiated melanocytes and UVSM. The number of melanosomes near the nucleus (distance from the nucleus < 1 μm) was significantly increased in UVSM **(Figure 2C, right panel)**, suggesting a defect in the melanosome transport process in senescent melanocytes. We confirmed these findings *in vivo* in skin biopsies from a donor with IGH. Senescent melanocytes are a characteristic of IGH, and p16^INK4A^-positive senescent melanocytes were identified frequently in the IGH skin **(Figure 2D)**. An EM analysis showed that in normal skin, melanosomes were mainly located in the peripheral dendrite region of the melanocytes. However, the IGH melanocytes showed shorter dendrites and perinuclear aggregation of melanosomes (**Figure 2E**). In addition to a different melanosome distribution, UVSM also showed an abnormal morphology of mitochondria with elongation and partial loss of cristae, which is a characteristic of senescent cells **(Figure S1)**. Melanosome transport dysfunction may arise during the excessive melanosome accumulation process in UVSM. Several types of motor-related genes, including kinesin and dynein, are involved in the melanin transport process **(Figure 2F),** and RNA sequencing data shows the down-regulation of these genes in the UVSM (**Figure 2G**). However, in order to transport melanin to the dendrites of the melanocytes, the most important protein is a tripartite complex consisting of *MYO5A, RAB27A,* and* MLPH*; down-regulation of the expression levels of these three genes was observed in UVSM. This was confirmed by western blot with significant decreases found in Myo5a, Rab27a and Mlph protein levels **(Figure 2H)**. All these findings indicate that senescent melanocytes are characterized by defective melanosome transport.

### Single-cell transcriptomics reveal glycolytic metabolism dysfunction in UVSM

**Figure 3:**
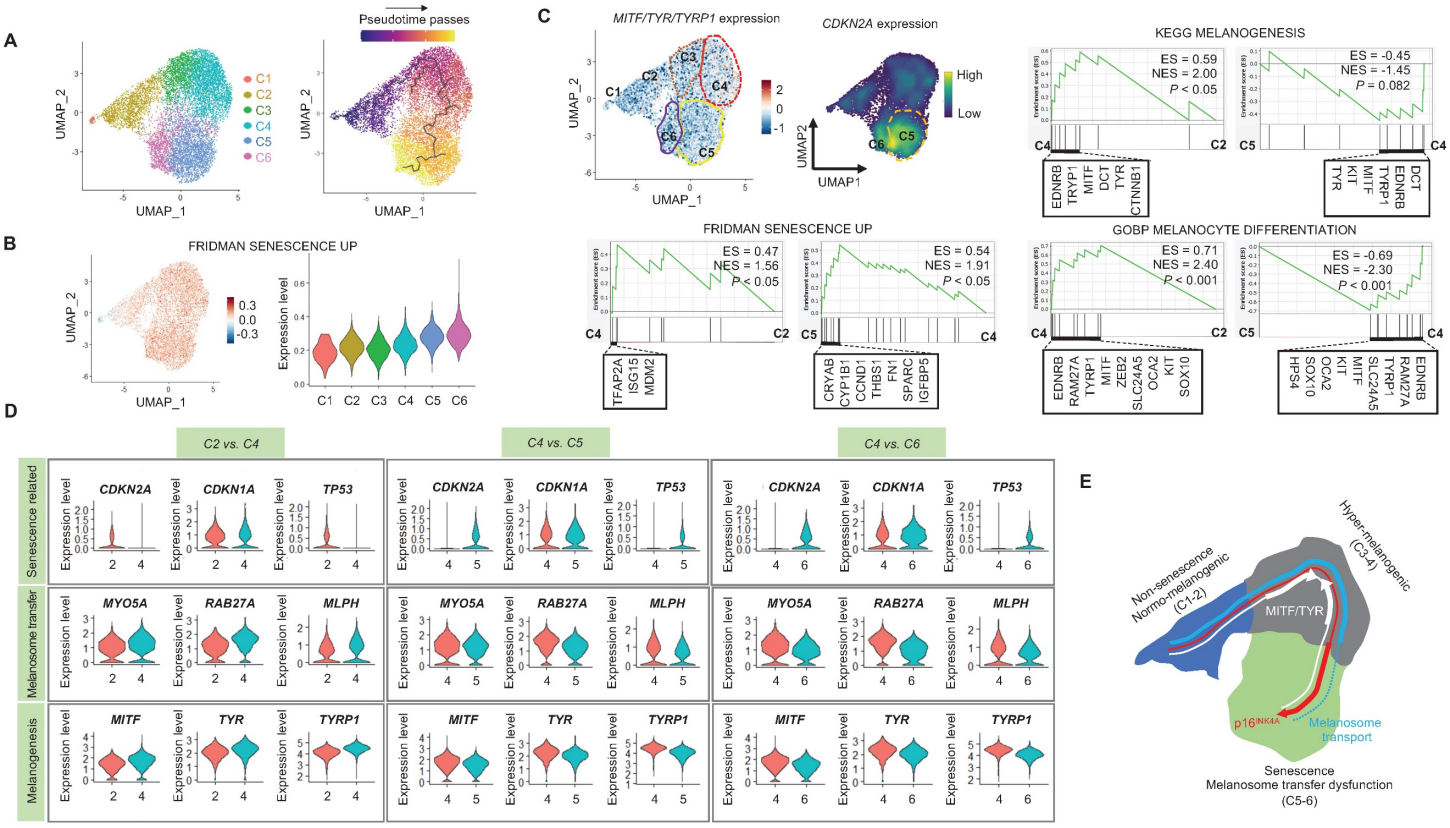
** Single-cell transcriptome analysis:** (A) Normal primary melanocytes were irradiated with UVB and cultured for ten days, with single-cell transcriptome data revealing six different subclusters, subsequently termed cluster 1 (C1) to cluster 6 (C6) according to the pseudotime that elapsed. (B) Senescent module scores using the “FRIDMAN senescence” gene set show a sequential increase from C1 to C6. (C) *MITF*, *TYR* and *CDKN2A* mRNA expression levels in single cell clusters (left upper panel). GSEA was performed using the “FRIDMAN senescence,” “KEGG melanogenesis” and “GOBP melanocyte differentiation” gene sets to demonstrate the sequential transition in gene expression levels from C2 to C4 and C4 to C5 (right upper and lower panel). (D) Violin plots showing the expression levels of mRNA-encoding senescence-related (*CDKN2A, CDKN1A, TP53*), melanosome transport (*MYO5A, RBA27A, MLPH*) and melanogenesis-related (*MITF, TYR, TYRP1*) gene sets to demonstrate the sequential transition in gene expression levels from C2 to C4, C4 to C5, and C4 to C6. (E) Schematic image of the gene expression changes in single cell clusters. Statistical significances were assessed using Mann-Whitney U test for numerical data (D) in independent two group comparison.

**Figure 4:**
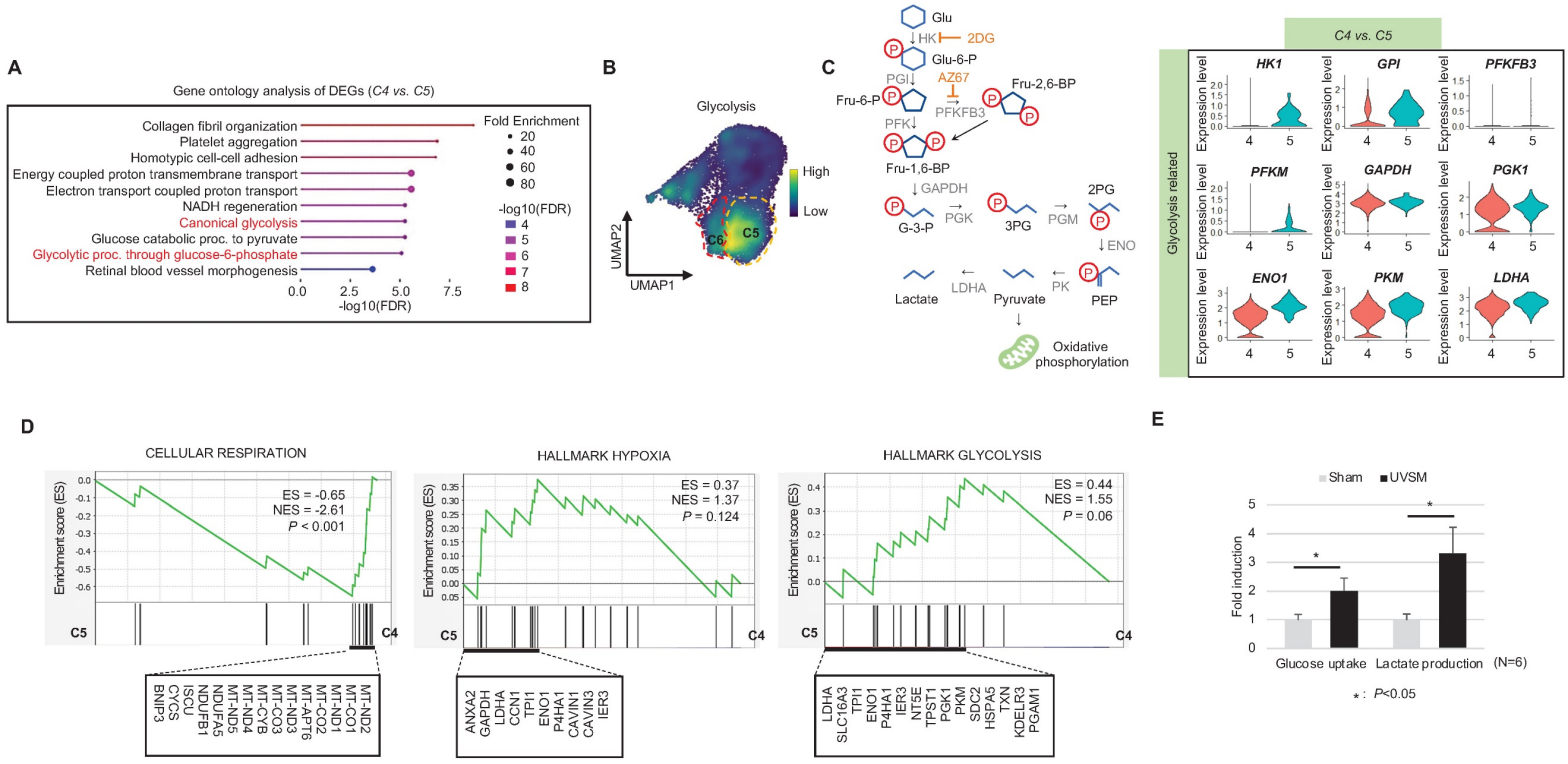
** Glycolytic metabolism dysfunction in senescent melanocytes:** (A) Gene ontology analysis results of differentially expressed genes between C4 and C5. (B) Glycolysis-related gene expression in single cell clusters. (C) A schematic image of the glycolysis pathway and corresponding inhibitors (left panel) and a violin plot analysis of glycolysis-related gene expression levels between C4 and C5 (right panel). (D) GSEA was performed using the “CELLULAR RESPIRATION,” “HALLMARK HYPOXIA” and “HALLMARK GLYCOLYSIS” gene sets to demonstrate the sequential transition in gene expression levels from C4 to C5. (E) Glucose uptake and lactate production outcomes were analyzed in sham-irradiated and UVB-induced senescent melanocytes. Statistical significances were assessed using Mann-Whitney U test for numerical data (D and E) in independent two group comparison.

When cells are exposed to sub-lethal doses of external stress, such as UV irradiation, they can exhibit various statuses in an *in vitro* culture dish. These statuses can range from nearly normal cells to stressed cells, and eventually senescent cells 22. Therefore, in this study, we aimed to investigate the changes in gene expression levels from nearly normal melanocytes to senescent melanocytes after UV irradiation. To achieve this at the single-cell level, we conducted a trajectory analysis based on single-cell RNA sequencing to track the fate of melanocytes by analyzing changes in their gene expression levels. Through this analysis, we finally sought to identify "senescent driver" genes in melanocytes. Firstly, we identified six different subclusters **(Figure 3A, left panel)**. Next, a pseudotime analysis of the cells was conducted, showing that senescent modules increased with the pseudotime trajectory **(Figure 3A, right panel)**. Based on the pseudotime, we termed the clusters C1 to C6 according to the sequential increase of the senescent module score **(Figure 3A-B)**. C4 was presumed to contain the most UV-reactive melanocytes, as indicated by the highest module score of melanogenesis (*MITF*, *TYR*, and *TYRP1*) **(Figure 3C)**. C5 and C6, on the other hand, showed a definite increase in the expression of the *CDKN2A* gene **(Figure 3C)**. We further analyzed the subclusters and found that senescence was sequentially increased from C2 to C4 and from C4 to C5, whereas melanogenesis and melanocyte differentiation were decreased at the point of transition from C4 to C5 **(Figure 3C)**. To specify the molecular changes that caused such an increase in senescent gene expression, we evaluated the DEGs between C2/C4, C4/C5, and C4/C6. Thus, we assigned C4 to cells in the process of senescence and C5 and C6 to fully senescent cells. Interestingly, genes related to melanogenesis, as observed in the above data, were downregulated in C5 and C6 as compared to C4, as were the melanosome transport-related genes of *MYO5A*, *RAB27A* and *MLPH* **(Figure 3D-E)**. These data show that fully senescent melanocytes and the melanocytes becoming senescent have different gene expression patterns. Therefore, the step from C4 to C5 could be the most important point to determine whether the melanocytes become senescent. By targeting this transition, we could inhibit or delay melanocyte senescence. Therefore, we analyzed the major gene expression changes during the transition from C4 to C5, focusing on the expression levels of glycolysis signaling-related genes as the major change factor **(Figure 4A)**. We were able to observe increased glycolysis gene set expressions in C5 and C6 **(Figure 4B)**. We also observed a significant decrease in aerobic respiration as well as a marked increase in glycolysis in C5 compared to C4 **(Figure 4C-D)**. C5 demonstrated the upregulation of multiple genes related to glycolysis, in this case *HK1* and *ENO1*, compared to C4 **(Figure 4C)**. To confirm the upregulation of the glycolysis pathway in senescent melanocytes, we investigated glucose uptake and lactate production in the UVSM cases. These results showed that UVSM consumed two times more glucose than sham-irradiated melanocytes and produced more lactate by 330% **(Figure 4E)**. Taken together, these results suggest that glycolysis is critical for the establishment of senescent in melanocytes and, in consequence, in senescence-associated defective melanosome transport.

### Regulation of glycolytic metabolism alleviates senescence in melanocytes

**Figure 5:**
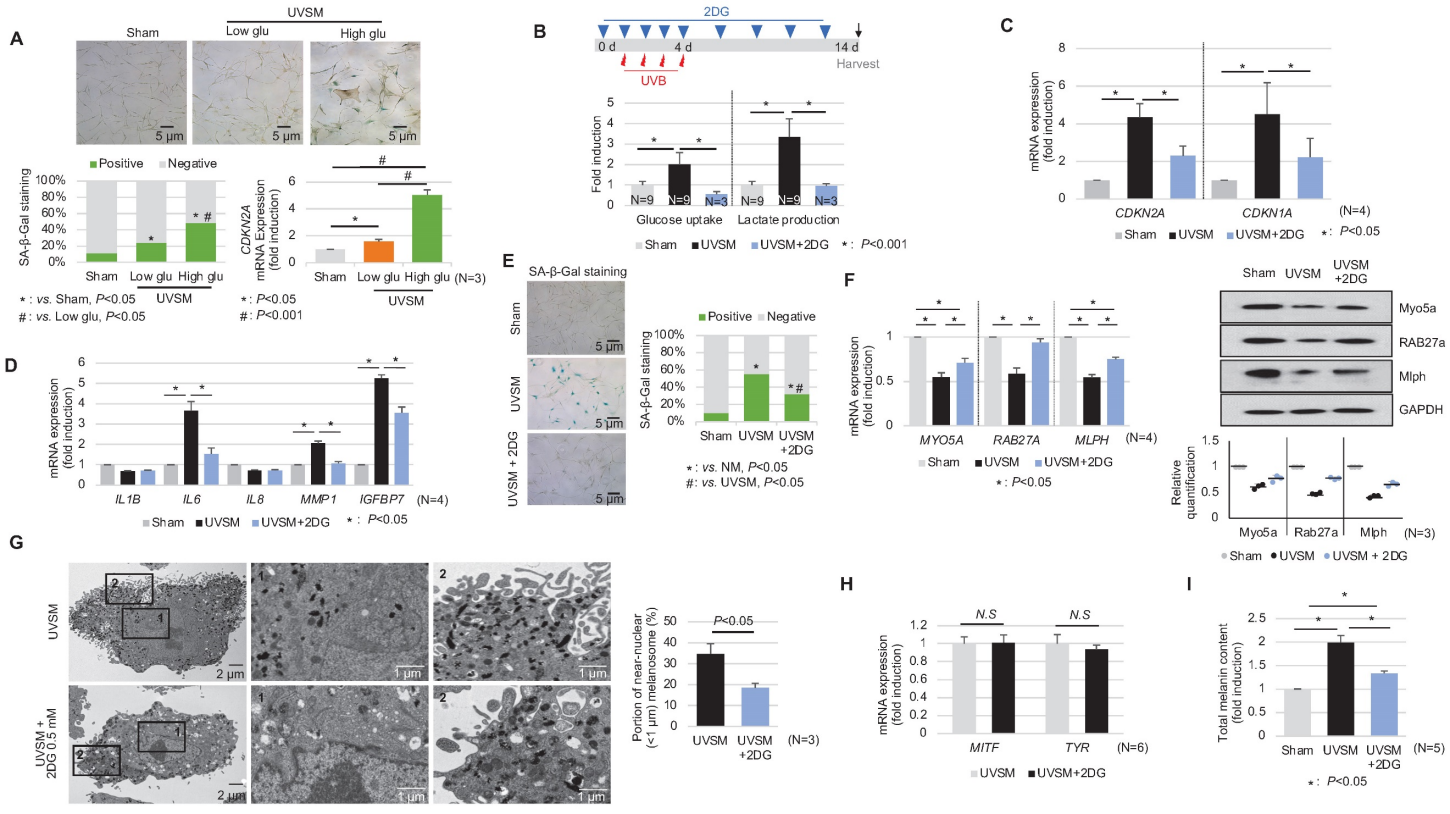
** Inhibition of glycolysis delays melanocytes senescence.** (A) Normal melanocytes were irradiated with UVB and maintained for 5 days in low- or high-glucose culture media and were then analyzed with SA-β-Gal staining and for mRNA expression. (B) Schematic image of the glycolysis inhibitor (2-DG) treatment schedule (upper panel). Glucose uptake and lactate production were analyzed in sham-irradiated and UVB-induced senescent melanocytes with or without 2-DG (lower panel). *CDKN1A, CDKN2A* (C) and SASPs (D) mRNA expression levels in sham-irradiated and UVB-induced senescent melanocytes with or without 2-DG. (E) SA-β-Gal staining in sham-irradiated and UVB-induced senescent melanocytes with or without 2-DG. Melanosome transport-related genes (*MYO5A, RAB27A* and *MLPH*) expression (F, left panel) and protein levels (F, right panel), melanogenesis-related genes (*MITF* and *TYR*) mRNA expression (G) and the total melanin content (H) were analyzed in UVB-induced senescent melanocytes with or without 2-DG. (I) UVB-induced senescent melanocytes and 2-DG treated senescent melanocytes were analyzed by EM (left panel). The melanosome distribution was analyzed and is presented here as a bar graph (right panel). Statistical significances were assessed using chi-square test for categorical data (A lower left and E; SA-β-Gal) and Mann-Whitney U test for numerical data (G and I) in independent two group comparison. One-way ANOVA with Tukey's post-hoc test was used for multiple comparisons among three groups (A lower right, B, C, D, F, and I), and only the significant results of post-hoc test between two group were indicated.

In addition to the single-cell RNA sequencing data presented above, we showed that normal human melanocytes cultured under high-glucose conditions (25 mM) show higher p16^INK4A^ expression levels, associated with SA-β-Gal positivity and *CDKN2A* mRNA upregulation **(Figure 5A)** and suggesting that alterations in cellular glucose metabolism act as a driving force for senescence induction in melanocytes. To evaluate the effect of a glycolysis blockage on melanocyte senescence, melanocytes were cultured for 24 hours with 0.5 mM 2-deoxy-D-glucose (2-DG), a well-known glycolysis inhibitor, prior to UV stimulation. 2-DG, a glucose analog, is known as a glycolysis blocker and as the first key gatekeeper of glycolysis 23. The inhibition of glycolysis, as indicated by reduced glucose consumption and lactate production in 2-DG-treated UVSM **(Figure 5B),** significantly downregulated the expression levels of the senescence markers of *CDKN2A* and *CDKN1A* **(Figure 5C)**. Furthermore, the SASP *IL-6*, *MMP1* and *IGFBP7* gene expression levels were also downregulated **(Figure 5D)**. In parallel, fewer SA-β-Gal-positive cells were observed **(Figure 5E)**, indicating that glycolysis inhibition alleviated cellular senescence in melanocytes. The melanosome transport function was maintained, i.e. melanosome transport-related genes *MYO5A, RAB27A* and *MLPH* and their protein levels were restored after the 2-DG treatment in UVSM **(Figure 5F)**. The 2-DG treated cells showed decreased melanin accumulation and fewer melanosomes near the nucleus **(Figure 5G)**. Although no significant changes in melanogenesis gene (*MITF* and *TYR)* expression levels were observed **(Figure 5H)**, melanin contents were reduced by the 2-DG treatment **(Figure 5I)**. We also tested another well-known glycolysis blocker, AZ67 (6-phosphofructo-2-kinase/fructose-2), a 6-bisphophatase 3 (PFKFB3) inhibitor, for its ability to delay melanocyte senescence 24. Although the senescence markers of *CDKN2A* and *CDKN1A* were downregulated, there were no significant changes in the expression levels of melanosome transport-related genes and melanogenesis-associated genes **(Figure S2)**.

## Discussion

The present study demonstrated that repetitive UVB irradiation can induce senescence in melanocytes, as shown by the significant increase in senescence-associated markers (SA-β-Gal, p16^INK4A^ and p21^Waf1^) in irradiated melanocytes. Moreover, senescent melanocytes exhibited the abnormal perinuclear aggregation of melanosomes, associated with a decrease in melanosome transport-related genes (*MYO5A, RAB27A* and* MLPH)*. We have also demonstrated the accumulation of melanin in senescent melanocytes which was not caused by an increase in melanin synthesis; this suggested that a melanosome transport dysfunction may be responsible for melanin accumulation. This finding is also thought to contribute to the further acceleration of cell senescence through increased ROS formation 1, 21.

These new findings not only characterized UV-induced senescent melanocytes but also allowed us to identify the turning point from a UV-induced reactive state to a UV-induced fully senescent state with key metabolic changes in melanocytes. This turning point may be a potential target to inhibit or delay senescence in melanocytes. A growing body of literature indicates that both senescence and the SASP are sensitive to cellular and organismal metabolic states, which in turn can drive phenotypes associated with metabolic dysfunction 25. Here, we suggest the occurrence of significant glycolytic metabolic imbalances related to senescence in melanocytes. Our single-cell RNA data indicated that senescent melanocytes undergo a shift of their major metabolism type from oxidative phosphorylation to glycolysis. Senescent melanocytes showed higher glucose consumption and lactate production levels. Conversely, high levels of glucose accelerated senescence in cultured melanocytes. These results suggested that alterations in cellular glucose metabolism act as the driving force behind senescence in melanocytes. It was shown that high glucose also drives senescence in skin fibroblasts and that this process is associated with low levels of SIRT3, a mitochondrial enzyme able to regulate acetylation levels in mitochondria 26. Interestingly, a recent system-biology-based analysis of photo-exposed human skin also found glycolysis-associated genes and proteins were increased in one's 50s compared to the 30s, suggesting imbalanced epidermal homeostasis in aging skin 27. That study suggested that a chronic inflammatory microenvironment when in one's 20s with younger aged skin leads to an imbalance in epidermal homeostasis, which results in a prematurely aged appearance during later life. However, it remains difficult to determine how glycolysis induces senescence in skin cells. Senescent cells should undergo metabolic reprogramming in order to maintain their viable growth-arrested state. Therefore, the causes and consequences of these metabolic shifts still require further investigations.

Based on the findings indicating that senescent cells exhibit metabolic alterations, targeting a higher glycolytic state of senescent cells is an alternative strategy that highlights the importance of metabolic reprogramming in regulating melanocyte senescence 25, 28. Although metabolic adaptation to glycolysis is a defense mechanism often used to escape or compensate for senescence in fully senescent cells 29, prior metabolic intervention could delay young melanocytes (including normal and reactive melanocytes as described in our single-cell analyses) from beginning the senescence process. Our data showed that blocking the glycolysis process using 2-DG led to delay the senescence of melanocytes and maintain the cell function such as melanosome transport. Indeed, studies of fibrotic diseases have revealed that glycolysis is the preferred energy source for fibroblasts and that the inhibition of glycolysis decreases the extracellular matrix production of fibroblasts, thereby decreasing fibrosis 30. Another study demonstrated that blocking the utilization of glucose by 2-DG induces a switch from senescence to apoptosis in human lung cancer cells 31. However, 2-DG has a broad impact on the induction of cell cycle arrest and apoptosis 32. Therefore, it remains unclear whether glycolytic inhibition efficiently regulates melanocyte senescence.

We speculate that melanosome transport dysfunction in senescent melanocytes is a phenotype of cellular senescence associated with impaired mitochondrial function, rather than being primarily attributed to glycolysis. In our study, there were no significant differences in the expression level of melanosome transport genes in young melanocytes cultured with 2-DG or high glucose **(Figure S3)**. In general, senescent cells are less efficient in ATP synthesis due to impaired mitochondria function and ATP production through anaerobic glycolysis 33. Our UVSM showed an abnormal morphology of mitochondria, characterized by elongation with partial loss of cristae **(Figure S1)**. We also found a significant decrease in ATP levels in senescent cells compared to young melanocytes **(Figure S4)**. This suggests that ATP synthesis is less efficient in senescent melanocytes. Because the melanosome transport proteins such as Mlph function using ATP 34, it is possible that melanosome transportation in senescent melanocytes is not efficient.

Melanocytes are pigment-producing cells that produce melanin in specific organelles, melanosomes, which are then transferred to keratinocytes. Melanin in keratinocytes is responsible for skin pigmentation as well as photoprotection 35. Our data showed that melanin accumulates in senescent melanocytes caused by defects in melanosome transport. This likely causes a reduced level of melanin in keratinocytes and thus less hypopigmentation of aging skin. Indeed, several studies have demonstrated the presence of senescent melanocytes in hypopigmented aging skin, such as the IGH type 9, 36. The number of p16^Ink4a^-expressing melanocytes was increased in hypopigmented IGH skin compared to perilesional normal skin 9. Indeed, the melanocytes from IGH patients are characterized by retracted or less-developed dendrites 36 and reduced melanin transfer to adjacent keratinocytes, resulting in the cytoplasmic accumulation of melanin. These results are in line with the features of our *in vitro* UVSM model. The formation, maturation and trafficking of melanosomes is crucial to pigmentation, and defects in this process lead to depigmented disorders such as Hermansky-Pudlak syndrome 37. Therefore, it appears to be feasible to assume that senescent melanocytes are key players in hypopigmented aging skin. More importantly, it has been suggested that senescent melanocytes may have a propagator role in skin aging via paracrine telomere dysfunctions of keratinocytes and fibroblasts 10, 12. Senescent melanocytes alone can express various inflammatory markers and SASPs, and these factors are known to induce skin aging by affecting non-senescent nearby cells and the extracellular matrix 12. This process subsequently results in the proliferation arrest of adjacent cells such as keratinocytes through CXCL3-dependent mitochondrial ROS and induces skin aging 12. Regarding the roles of senescent melanocytes in skin aging, further validation studies that cultivate senescent melanocytes with keratinocytes or with skin equivalents are necessary.

## Materials and Methods

### Cell culture

Primary normal human melanocytes (HEMn-MP, Gibco, C1025C) at passages 3-7 obtained from moderately pigmented neonatal foreskin were incubated in Medium-254 (M254; Gibco) supplemented with Human Melanocyte Growth Supplement (HMGS; Gibco).

### *In vitro* models of senescent melanocytes

For UV-irradiated senescent melanocytes, primary human melanocytes were washed once with Dulbecco′s Phosphate Buffered Saline (DPBS) containing calcium chloride and magnesium chloride (Gibco) and placed in fresh DPBS. The cells were irradiated with 60 mJ/cm^2^ UVB (maximum peak 313 nm) every day for 4 consecutive days using LZC-1 photoreactor system (Luzchem Research Inc, Ontario, Canada). After irradiation, the cells were maintained in F12 medium (Gibco, Thermo Fisher Scientific, Waltham, MA) supplemented with 10% heat-inactivated fetal bovine serum (FBS; Gibco), 1% Penicillin/streptomycin (Gibco), 24 μg/ml 3-isobutyl-1-methylxanthine (Sigma, St. Louis, MO), 1.2 ng/ml basic fibroblast growth factor (Sigma, St. Louis, MO), and 0.1 μg/ml cholera toxin (List biological, Campbell, CA) for 10 days.

### Senescence associated β-galactosidase (SA-β-Gal) staining

Cells were fixed with 4% formalin for 15 minutes and then incubated with senescence associated β-galactosidase solution (X-gal, 1 mg/ml; citric acid/sodium phosphate, pH 5.8, 40 mM; potassium ferrocyanide, 5 mM; potassium ferricyanide, 5 mM; NaCl, 150 mM; and MgCl_2_, 2 mM) for 12 hours at 37 °C. After washing with PBS, senescence-associated β-galactosidase-positive cells were analyzed under light microscopy.

### Heterochromatin foci

For the heterochromatin foci analysis, cells were fixed with 4% paraformaldehyde for 15 minutes at room temperature. The cells were stained with DAPI for 10 minutes, after which the heterochromatin foci were analyzed with a fluorescence microscope (Axiovert 200M, Carl Zeiss Macroscopy GmbH, Jana, Germany) at the Three-Dimensional Immune System Imaging Core Facility of Ajou University.

### Real-time PCR analysis

Total cellular RNA was extracted using the RNeasy Mini kit (Qiagen, Hilden, Germany) and the cDNA was obtained using a SuperScript III Reverse Transcriptase Kit (Invitrogen). Real-time PCR was carried out with iQ SYBR Green Supermix (Bio-Rad) using the following conditions: initial activation at 95 °C for 5 minutes, followed by 40 cycles at 95 °C for 15 seconds and 60 °C for 1 minute. The primers used for the real-time PCR are as follows: human 18S: 5′-CGGCTACCACATCCAAGGAA-3′ and 5′-GCTGGAATTACCGCGGCT-3′; MITF: 5′-AGAACAGCAACGCGCAAAAGAAC-3′ and 5′-ATGATCCGATTCACCAAATCTG-3′; tyrosinase: 5′-CACCACTTGGGCCTCAATTTC-3′ and 5′-AAAGCCAAACTTGCAGTTTCCAC-3′; p16^INK4A^: 5′-GAGCAGCATGGAGCCTTC-3′ and 5′-GGCCTCCGACCGTAACTATT-3′; p21^Waf1^: 5′-AGGTGGACCTGGAGACTCTCAG-3′ and 5′-TCCTCTTGGAGA AGATCAGCCG-3′; Myosin 5A: 5′-AGTCTCTGTGTCGTTCATTCG-3′ and 5′-CCTGTATGTAAACTCACG GTA-3′; RAB27A: 5′-TGGAGGACCAGAGAGTAGTGAAA-3′ and 5′-AGTTTCAAAGTAGGGGATTCCA-3′; MLPH: 5′-GCCCATCAAACCAACAGACA-3′ and 5'-TCCTCTGGTCTCTTGCCAAG-3′; IL-1β: 5′-AAATACCTGTGGCCTTGGGC-3′ and 5′-TTTGGGATCTACACTCTCCAGCT-3′; IL-6: 5′-CCACACAGACAGCCACTCAC-3′ and 5′-AGGTTGTTTTCTGCCAGTGC-3′; MMP1: 5′-AAGCGTGTGACAGTAAGCTA-3′ and 5′-AACCGGACTTCATCTCTG-3′; MMP3: 5′-GGACAAAGGATACAACAGGGACCA-3′ and 5′-GAACCGAGTCAGGTCTGTGAGTG-3′; IL-8: 5'-ATGACTTCCAAGCTGGCCGTGGCT-3' and 5'-TCTCAGCCCTCTTCAAAAACTTCT-3'; IGFBP7: 5'-GGCATGGAGTGCGTGAAGAG-3' and 5'-CTTGCTGACCTGGGTGATGG-3'; CCL2: 5'-CTCTGCCGCCCTTCTGTG-3' and 5'-TGCATCTGGCTGAGCGAG-3'; CCL3: 5'-TGCAACCAGTTCTCTGCATC-3' and 5'-AATCTGCCGGGAGGTGTA-3'; CCL20: 5'-ATGTGCTGTACCAAGAGTTT-3' and 5'-CAAGTCTGTTTTGGATTTGC-3'; CCL8: 5'-ATGACTTCCAAGCTGGCCGTGGCT-3' and 5'-TCTCAGCCCTCTTCAAAAACTTCT-3'; CXCL1: 5'-AGCTTGCCTCAATCCTGCAT-3' and 5'-CCTCTGCAGCTGTGTCTCTC-3'; CCL5: 5'-ATGAAGGTCTCCGCGGCAGC-3' and 5'-CTAGCTCATCTCCAAAGAGT-3'.

### Western blotting

Cells were lysed in RIPA buffer (1% NP-40; 150 mM NaCl; 10 mM Tris-HCl, pH 8.0; and 1 mM EDTA) with a complete protease inhibitor (Sigma). The proteins were separated by SDS-polyacrylamide gel and then transferred to a polyvinylidene fluoride membrane (Millipore, Burlington, MA). The antibodies against MITF (ab12039) and RAB27 (ab55667) were obtained from Abcam (Cambridge, United Kingdom); Tyrosinase (sc7833) was from Santacruz Biotechnology (Dallas, TX); Myosin 5A (NBP2-93397) was from Novus Biologicals (Centennial, CO); Mlph was from proteintech (Chicago, IL).

### Melanin content and DOPA oxidase activity of tyrosinase

Melanocytes were lysed with a 0.1 M phosphate buffer (pH 6.8) containing 1% Triton X-100 and a protease inhibitor cocktail (Roche, Basel, Switzerland). The supernatants were measured to determine the protein concentration using the Lowry assay system. Pellets were solubilized in 100 μL of 1 M NaOH for 3 hours at 60 °C, and the absorbance was measured at 490 nm to determine the melanin content relative to a standard curve using synthetic melanin (Sigma). For the DOPA oxidase activity of tyrosinase, each sample was incubated with 2 mM L-DOPA (Sigma) in a 0.1 M phosphate buffer (pH 6.8) for 90 minutes at 37 °C. After incubation, the DOPA oxidase activity was measured at 490 nm with an ELISA reader (Bio-Rad, Hercules, CA).

### Electron microscopic observations

The samples were dehydrated in a series of ethanol solutions, infiltrated with propylene oxide, embedded in Epon Mixture (Polysciences, PA, USA), and sequentially incubated at 60 °C (24 hours). The sample blocks were sectioned using an ultramicrotome (Reichert-Jung, Bayreuth, Germany). For contrast staining, sections were stained with 6% uranyl acetate (Electron Microscopy Sciences, PA) for 20 min and lead citrate (Thermo Fisher Scientific) for 10 min. Images were obtained using a SIGMA500 transmission electron microscope (Carl Zeiss Microscopy GmbH, Jena, Germany) at the Three-Dimensional Immune System Imaging Core Facility of Ajou University.

### Immunohistochemical (IHC) analysis

Immunohistochemical staining was performed on 4% paraformaldehyde-fixed, paraffin-embedded sections. The histological sections (4 μm) were deparaffinized and rehydrated in xylene and ethanol bathes. Sections were then incubated in 0.05% trypsin in Tris-buffered saline for 20 minutes at 37 °C for antigen retrieval. The primary antibodies used were as follows: anti-MART-1 (MS-799-P, dilution 1:100, Neomarker, Fremont, CA) to visualize melanocytes, and anti-p16^INK4A^ rabbit monoclonal antibodies (1:100, 725-4713, Ventana Medical Systems, Inc., Oro Valley, AZ) to visualize senescent cells.

### Single-cell transcriptomic analysis

Single-cell suspensions were obtained from live UV-stimulated melanocytes as described above. Single-cell RNA sequencing was performed using a Chromium Single Cell 3' V3 Reagent kit (10X Genomics, Pleasanton, CA) according to the manufacturer's protocol. Next-generation sequencing was performed using an Illumina HiSeq X Ten System (San Diego, CA USA) for 10,000 cells per sample. Sequencing results were converted into FASTQ files using Cell Ranger (10X Genomics). Samples were aligned using the human genome GRCh38 as a reference. Matrices were loaded into Seurat (v.3) for data analysis, and R (v. 3.6.1) was used for statistical analyses 38. Cells with an RNA count too low < 200 or mitochondrial gene expression too high (> 15%) were excluded. Data were normalized using the NormalizeData function. Variance of gene expression was checked using the FindVariableFeature function. Data dimensions were reduced using principal component (PC) analysis (PCA) to 20 significant PCs for each sample. Data were clustered using the FindNeighbors and FindClusters functions at various resolutions. RunUMAP was used to visualize the selected PCs. Data were aligned and normalized for the number of genes per cell and mitochondrial gene content using the Seurat workflow. Cluster markers were identified using the FindMarkers function. Pseudotime-trajectory calculation was performed using Monocle3 (v.0.2.3.0) with ribosomal genes excluded removed from the gene expression matrix.

### Gene set enrichment analysis (GSEA)

GSEA was performed using the GSEA v.4.3.2 software. Rank data was extracted from all the DEG between sham and UVSMA in bulk RNA sequencing. In single cell analysis data, we used the DEG with more than 0.25 of log_2_FC as the rank data. All gene set files for this analysis were obtained from molecular signatures database of GSEA website (http://www.gsea-msigdb.org/gsea/msigdb). Enrichment score (ES), normalized ES (NES), and adjusted *P*-value were extracted for each gene set after 1,000 times gene set permutations, and enrichment map was visualized to show the GSEA results.

### Glycolytic flux analysis

Intracellular glycolytic flux was quantitatively estimated by measuring glucose uptake and lactate excretion in cell culture media 39. Conditioned media from normal melanocytes and UVSMs were collected, and the concentrations of glucose and lactate were measured in the media using the YSI 7100 MBS Multiparameter Biochemistry Analyzer (YSI Inc., Yellow Springs, OH) according to the manufacturer's instructions. We also measured their concentration of fresh F12 not incubated with cells to calculate the change of glucose and lactate concentration in the process of intracellular metabolism.

### Glycolysis inhibition

For glycolysis inhibition, primary human melanocytes were treated with 0.5 mM 2-deoxy-D-glucose (2-DG, Sigma, St. Louis, MO) or 10 nM AZ67 (Tocris Bioscience, Bristol, UK) before (day 0) and during UVB irradiation (days 1 to 4). Cells were maintained in F12 medium and were treated again with 2-DG or AZ67 following the UV irradiation (days 6, 8, 10, and 12) before harvest (day 14).

### Measurement of ATP levels in melanocytes

Intracellular ATP level was measured using the ENLIGHTEN ATP assay system according to the manufacturer's instructions (Promega, Madison, WI). Briefly, ATP was extracted from the melanocytes (10^5^ cells) using 2.5% TCA and neutralized with 0.1 M Tris-acetate buffer pH 7.75. Following addition of an equal volume of Luciferase/Luciferin Reagent (Promega), luminescence was measured as readout for ATP levels.

### Statistical analysis

Data were presented as the mean ± standard deviation (SD) of independent measurements. Statistical significances were assessed using chi-square test for categorical data and Mann-Whitney U test for numerical data in independent two group comparison. One-way analysis of variance (ANOVA) was used to compare means between three groups. Furthermore, Tukey's post hoc analysis was conducted to determine which specific group means differ from each other when the significant result from ANOVA were found, and only the significant results of post-hoc test between two group were indicated in the Figure. The results were considered statistically significant when the two-tailed p-value was < 0.05. IBM SPSS ver. 25 (IBM, Armonk, NY) was used for all statistical analyses.

## Supplementary Material

Supplementary figures.
