# Supplementary material for: Senescent melanocytes driven by glycolytic changes are characterized by melanosome transport dysfunction
Source: Theranostics. 2023 Jul 3;13(12):3914–24. doi: 10.7150/thno.84912 (PMC10405833; doi:10.7150/thno.84912)
Supplement: Supplementary file 1 — Supplementary figures. [file thnov13p3914s1.pdf]

Figure S1. EM analysis. Sham-irradiated and UVB-induced senescent melanocytes were analyzed by EM. Arrows indicate abnormal mitochondria.

Figure S2. PFKB3 inhibition by AZ67 did not influence melanocyte senescence. Glucose uptake, lactate production, senescence-related (*CDKN1A* and *CDKN2A*), melanosome transport-related (*MYO5A, RAB27A* and *MLPH*) and melanogenesis-related (*MITF* and *TYR*) gene mRNA expression levels in sham-irradiated and UVB-induced senescent melanocytes with or without AZ67. One-way ANOVA with Tukey's post-hoc test was used for multiple comparisons among three groups, and only the significant results of post-hoc test between two group were indicated.

Figure S3. Normal melanocytes (NM) were maintained for 24 hours in high-glucose (25 mM) culture media (A) and standard glucose culture media with 2-DG (B), respectively, and were then analyzed for mRNA expression of melanosome transport-related genes (*MYO5A*, *RAB27A* and *MLPH*). Statistical significances were assessed using Mann-Whitney U test for independent two group comparison.

Figure S4. Intracellular ATP level were analyzed in sham-irradiated and UVB-induced senescent melanocytes (each group consisted of 10^5 cells). Statistical significances were assessed using student t-test for independent two group comparison.

Figure S1

UV-induced senescent melanocyte

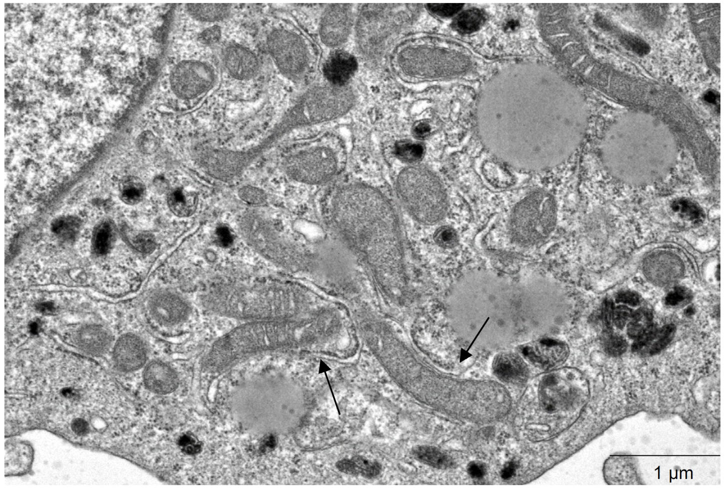

Figure S2

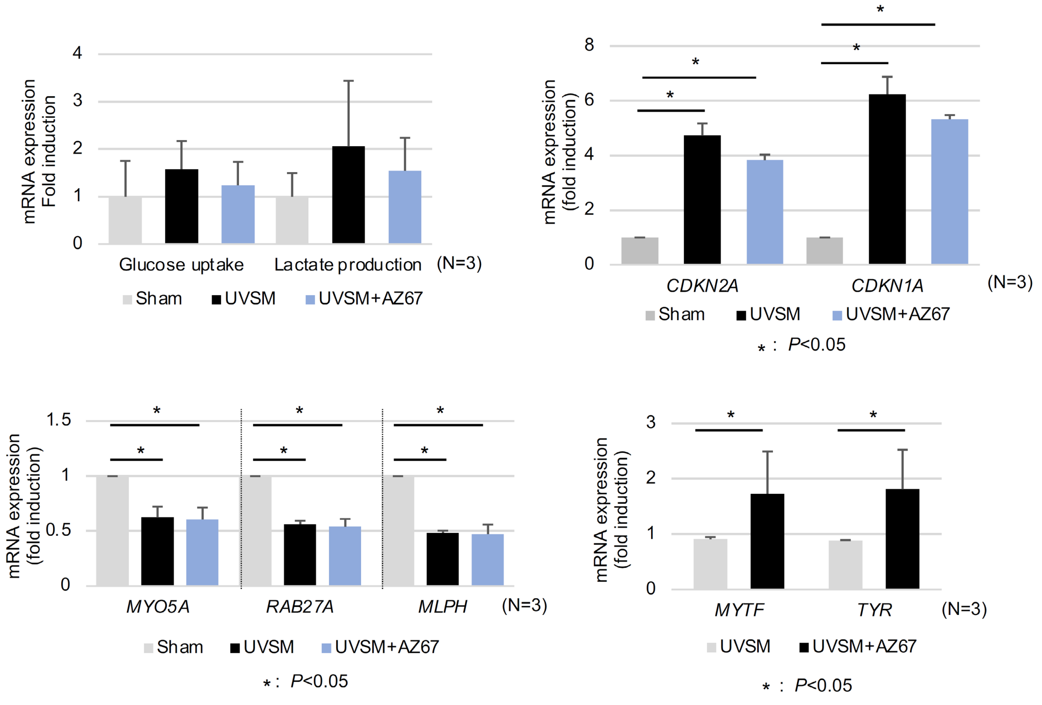
mRNA expression Fold induction
4
3
2
1
0
Glucose uptake
Lactate production
(N=3)
Sham
UVSM
UVSM+AZ67
mRNA expression (fold induction)
8
6
4
2
0
*
*
*
*
CDKN2A
CDKN1A
(N=3)
Sham
UVSM
UVSM+AZ67
*: P<0.05
mRNA expression (fold induction)
1.5
1
0.5
0
*
*
*
*
*
*
MYO5A
RAB27A
MLPH
(N=3)
Sham
UVSM
UVSM+AZ67
*: P<0.05
mRNA expression (fold induction)
3
2
1
0
*
*
MYTF
TYR
(N=3)
UVSM
UVSM+AZ67
*: P<0.05

Figure S3

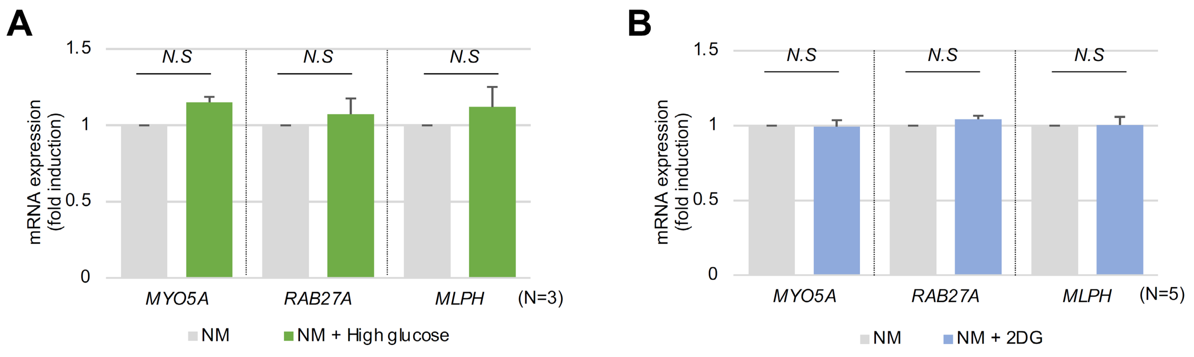
A
1.5
1
0.5
0
mRNA expression
(fold induction)
N.S
N.S
N.S
MYO5A
RAB27A
MLPH
(N=3)
NM
NM + High glucose
B
1.5
1
0.5
0
mRNA expression
(fold induction)
N.S
N.S
N.S
MYO5A
RAB27A
MLPH
(N=5)
NM
NM + 2DG

Figure S4

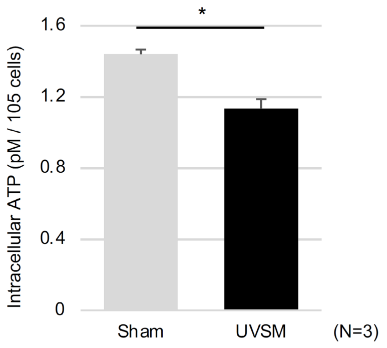

* : *P*<0.05
